# Contribution of Bone Marrow-Derived Hematopoietic Stem/Progenitor Cells to the Generation of Donor-Marker^+^ Cardiomyocytes *In Vivo*

**DOI:** 10.1371/journal.pone.0062506

**Published:** 2013-05-07

**Authors:** Mitsuhiro Fukata, Fumihiko Ishikawa, Yuho Najima, Takuji Yamauchi, Yoriko Saito, Katsuto Takenaka, Kohta Miyawaki, Hideki Shimazu, Kazuya Shimoda, Takaaki Kanemaru, Kei-ichiro Nakamura, Keita Odashiro, Koji Nagafuji, Mine Harada, Koichi Akashi

**Affiliations:** 1 Department of Medicine and Biosystemic Science, Kyushu University Graduate School of Medical Science, Fukuoka, Japan; 2 Laboratory for Human Disease Models, RIKEN Research Center for Allergy and Immunology, Yokohama, Japan; 3 Department of Gastroenterology and Hematology, Faculty of Medicine, Miyazaki University, Miyazaki, Japan; 4 Morphology Core, Kyushu University, Fukuoka, Japan; 5 Second Department of Anatomy, Kurume University School of Medicine, Kurume, Japan; 6 Division of Hematology and Oncology, Department of Medicine, Kurume University School of Medicine, Kurume, Japan; 7 Center for Cellular and Molecular Medicine, Kyushu University Hospital, Fukuoka, Japan; Centro Cardiologico Monzino, Italy

## Abstract

**Background:**

Definite identification of the cell types and the mechanism relevant to cardiomyogenesis is essential for effective cardiac regenerative medicine. We aimed to identify the cell populations that can generate cardiomyocytes and to clarify whether generation of donor-marker^+^ cardiomyocytes requires cell fusion between BM-derived cells and recipient cardiomyocytes.

**Methodology/Principal Findings:**

Purified BM stem/progenitor cells from green fluorescence protein (GFP) mice were transplanted into C57BL/6 mice or cyan fluorescence protein (CFP)-transgenic mice. Purified human hematopoietic stem cells (HSCs) from cord blood were transplanted into immune-compromised NOD/SCID/IL2rγ^null^ mice. GFP^+^ cells in the cardiac tissue were analyzed for the antigenecity of a cardiomyocyte by confocal microscopy following immunofluorescence staining. GFP^+^ donor-derived cells, GFP^+^CFP^+^ fused cells, and CFP^+^ recipient-derived cells were distinguished by linear unmixing analysis. Hearts of xenogeneic recipients were evaluated for the expression of human cardiomyocyte genes by real-time quantitative polymerase chain reaction. In C57BL/6 recipients, Lin^−/low^CD45^+^ hematopoietic cells generated greater number of GFP^+^ cardiomyocytes than Lin^−/low^CD45^−^ mesenchymal cells (37.0+/−23.9 vs 0.00+/−0.00 GFP^+^ cardiomyocytes per a recipient, P = 0.0095). The number of transplanted purified HSCs (Lin^−/low^Sca-1^+^ or Lin^−^Sca-1^+^c-Kit^+^ or CD34^−^Lin^−^Sca-1^+^c-Kit^+^) showed correlation to the number of GFP^+^ cardiomyocytes (P<0.05 in each cell fraction), and the incidence of GFP^+^ cardiomyocytes per injected cell dose was greatest in CD34^−^Lin^−^Sca-1^+^c-Kit^+^ recipients. Of the hematopoietic progenitors, total myeloid progenitors generated greater number of GFP^+^ cardiomyocytes than common lymphoid progenitors (12.8+/−10.7 vs 0.67+/−1.00 GFP^+^ cardiomyocytes per a recipient, P = 0.0021). In CFP recipients, all GFP^+^ cardiomyocytes examined coexpressed CFP. Human troponin C and myosin heavy chain 6 transcripts were detected in the cardiac tissue of some of the xenogeneic recipients.

**Conclusions/Significance:**

Our results indicate that HSCs resulted in the generation of cardiomyocytes via myeloid intermediates by fusion-dependent mechanism. The use of myeloid derivatives as donor cells could potentially allow more effective cell-based therapy for cardiac repair.

## Introduction

Modification of regenerative capacity in injured heart could be potentially alternative to conventional therapy for treating patients suffering from heart failure [1]–[7]. Based on the promising results in rodents [3], [4], clinical trials of cellular therapy using bone marrow (BM) cells for ischemic heart disease patients have been designed. In many of clinical trials for improving the function of cardiac recovery, some favorable results were obtained following injection of BM mononuclear cells (MNCs) [2], [5]–[7]. However, careful examination needs to be performed in basic research because cell fate and the effects of transplanted cells are not fully unveiled [8].

BM contains heterogeneous cell populations including at least two distinct stem cells, hematopoietic stem cells (HSCs) and mesenchymal stem cells (MSCs) [9], and various progenitors of myeloid and lymphoid lineages. Both HSCs and MSCs have been reported to acquire the phenotype of cardiomyocytes in syngeneic or xenogeneic recipients [4], [10]–[13]. However, quantitative analysis of regenerative capacity by each stem fraction has not been performed in the identical transplantation setting.

One proposed mechanism for the phenotypic change of BM-derived cells to tissue-specific cells is cell fusion. Since the original report of spontaneous cell fusion between BM cells and embryonic stem cells [14], it has become apparent that not only some BM-derived cells in the heart and other selective tissues are the consequences of cell fusion at least in part [10], [12], [15], but also fused BM-derived cells can be reprogrammed to express tissue specific genes [16], [17]. On the other hand, BM cells have been reported to generate non-hematopoietic cells in certain tissues without fusion requirement [18], [19] although cell fate conversion from HSCs themselves directly to cardiomyocytes has questioned in several studies [10], [20], [21].

To improve the efficiency of cardiac functional restoration and to minimize adverse effects of cell-based therapy using BM cells, the cell type with the greatest contribution to cardiomyogenesis and mechanisms underlying altered cardiac function need to be clarified *in vivo*. In this study, we examined cardiomyogenic potential of BM cells following syngeneic BM transplantation to identify the cell population in BM that possesses cardiomyogenic potential and to clarify whether cardiomyogenesis by BM-derived cells require cell fusion with recipient cardiomyocytes. Furthermore, we adopted xenogeneic transplantation system to unveil genetic sequences of donor-derived cardiomyocytes through discrimination of the donor gene from the recipient gene. To fully exploit the capacity of stem cells, we employed newborns as recipients, in which age-related decline of regenerative capacity can be restored by environmental factors [22].

## Results

### Determination of Cell Fate in Cardiac Tissue of Recipient Mice Transplanted with Syngeneic BM Cells

**Figure 1:**
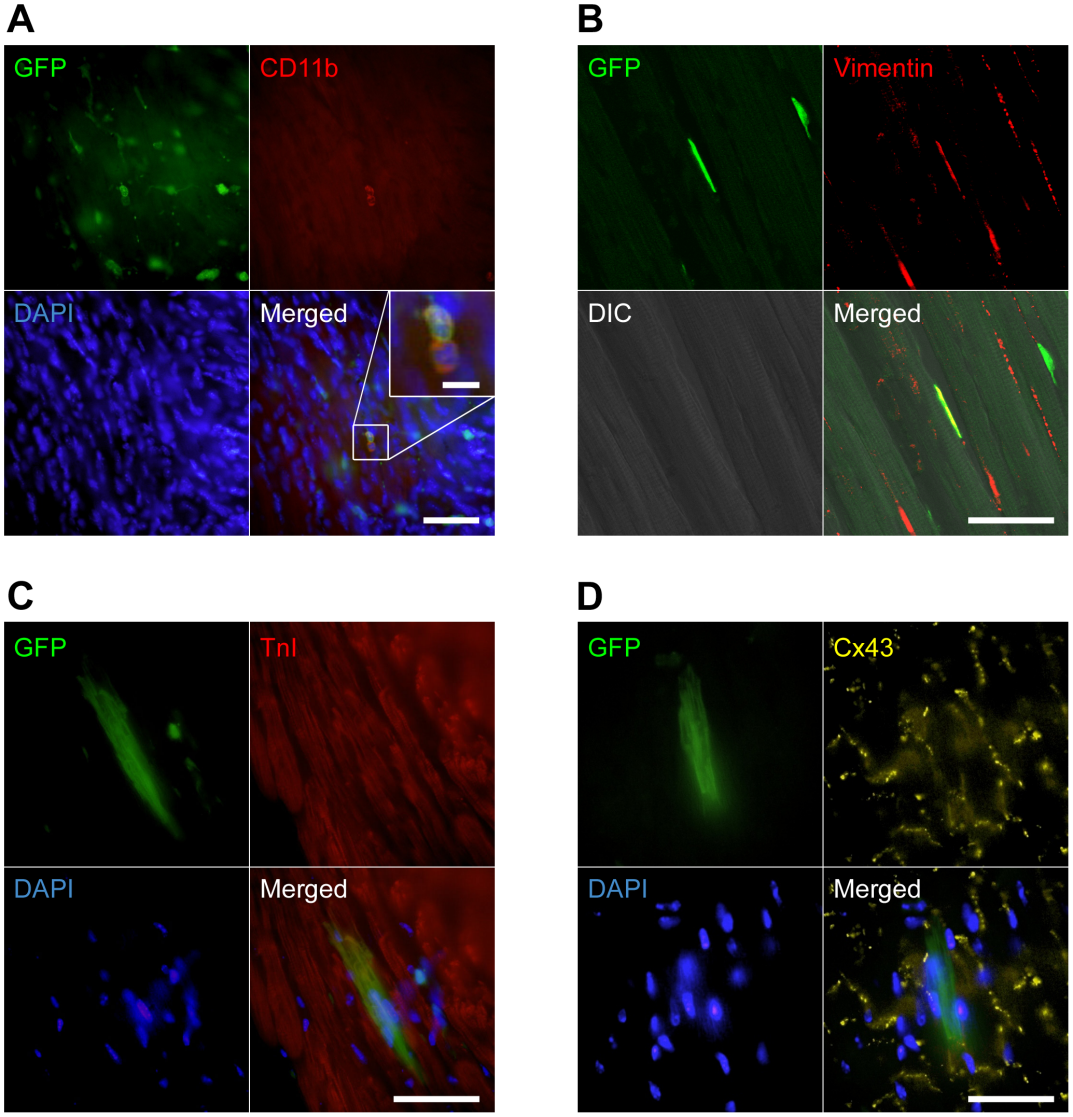
Characterization of donor BM-derived GFP^+^ cells in injured heart. (A) Representative image of CD11b-expressing GFP^+^ myeloid cells. GFP^+^ hematopoietic cells in recipient cardiac tissue appeared in small and round shape. Cardiac section was stained with anti-CD11b (red, Cy3) and DAPI (blue). Inset: high magnification of GFP^+^CD11b^+^ cells. (B) Representative image of a vimentin-expressing GFP^+^ fibroblast. Cardiac section was stained with anti-vimentin (red, Cy3). The fibroblast was present adjacent to striated cardiomyocytes in differential interference contrast (DIC) image. (C and D) Representative images of a TnI- (C) or Cx43- (D) expressing GFP^+^ striated cardiomyocyte. Cardiac sections were stained with anti-TnI (C; red, Cy3), anti-Cx43 (D; yellow, Cy5), and DAPI (blue). Cardiac sections are from recipients transplanted with unfractionated BM cells. All merged images were obtained from the same confocal plane. Scale bars = 50 µm, (A)-inset 10 µm.

We first created an *in vivo* model for evaluating cell fate of BM cells in cardiac tissue by injecting 10^7^ unfractionated green fluorescence protein (GFP) mouse BM cells into irradiated newborn C57BL/6 mice, followed by ventricular puncture. In the recipients, we detected GFP^+^ cells preferentially located adjacent to the injured cites. GFP^+^ cells in recipient cardiac tissues included CD45^+^ or CD11b^+^ hematopoietic cells (Figure 1A), vimentin^+^ fibroblasts (Figure 1B), cardiac troponin I (TnI)^+^ and/or Connexin 43 (Cx43)^+^ cardiomyocytes (Figure 1C and 1D) indicating that the system could be used for analyzing differentiative and regenerative properties of donor stem/progenitor cells. Cardiomyocytes were counted by their specific intracellular striated structure and longer diameter compared with hematopoietic cells. Immunofluorescence studies confirmed that the counted cells were cardiomyocytes as evidenced by the expression of TnI. Since the frequencies of GFP^+^ cardiomyocytes were similar in recipients transplanted with total BM cells or in those transplanted with Lin^−/low^ MNCs, we postulated that the cardiomyogenic cells in BM are enriched in immature Lin^−/low^MNCs.

### BM-derived Cardiomyocytes Originate from the Hematopoietic Lineage

We next determined the contribution of HSCs and MSCs, two already-defined stem cells in BM, to the generation of GFP^+^ cardiomyocytes. Multi-lineage differentiation capacities of HSCs included in the CD45^+^ fraction and MSCs included in the CD45^−^ fraction were confirmed by the development of lymphoid and myeloid cells *in vivo* and the induction of osteoblasts and adipocytes *in vivo* and *in vitro*, respectively (see Figure S1). To compare the cardiomyogenic abilities, 1.8×10^6^–4.4×10^7^ Lin^−/low^CD45^+^ cells and 3.6×10^5^–2.5×10^6^ Lin^−/low^CD45^−^ cells from GFP mice were transplanted into syngeneic recipients. At 4–6 weeks after transplantation, GFP^+^ cardiomyocytes were detected only in recipients transplanted with Lin^−/low^CD45^+^ cells (n = 6). By contrast, GFP^+^ cardiomyocytes were not detected in recipients transplanted with Lin^−/low^CD45^−^ cells (n = 4; P = 0.0095 Lin^−/low^CD45^+^ versus Lin^−/low^CD45^−^; Table 1) nor in the recipient transplanted with purified GFP^+^Lin^−^CD45^−^ cells. These findings suggest that donor-derived cardiomyocytes following BM transplantation originate from the hematopoietic lineage at least in this transplantation setting.

**Table 1:** The number of donor-derived cardiomyocytes after transplantation of purified BM cells.

Donor cell type		Cell dose	n	Days after injection	BM %GFP^+^	PB %GFP^+^	GFP^+^ cardiomyocytes*
Hematopoietic cell	Lin^−/low^CD45^+^	0.2–4.4×10^7^	6	25–46	86.7+/−13.9	90.6+/−5.6	37.0+/−23.9^a^
Mesenchymal cell	Lin^−/low^CD45^−^	0.4–2.5×10^6^	4	25–39	0.35+/−0.39	0.03+/−0.05	0.00+/−0.00^a^
HSC	Lin^−/low^Sca-1^+^	10^6^	5	29–34	83.2+/−6.6	96.4+/−2.9	33.9+/−16.0
		10^5^	5	29–39	54.8+/−17.8	75.5+/−17.0	21.8+/−10.7
		10^4^	5	28–39	3.0+/−1.6	6.0+/−7.6	4.2+/−3.4
		10^3^	5	28–39	0.59+/−0.40	1.6+/−2.5	1.2+/−1.1
	LSK	1.0–1.8×10^5^	6	27–44	76.6+/−17.7	89.1+/−17.0	33.6+/−43.1
		1.0–4.0×10^4^	8	31–52	36.2+/−25.1	45.0+/−32.8	9.3+/−10.3
		1.0–2.0×10^3^	6	31–52	25.5+/−23.3	38.2+/−27.6	2.4+/−2.0
	CD34^−^LSK	10^3^	2	28–50	66.8+/−27.3	67.3+/−23.6	12.5+/−5.0
		10^2^	3	31–57	6.7+/−10.9	15.5+/−14.9	3.4+/−2.5
		10	7	213–354	0.19+/−0.13	0.05+/−0.05	0.14+/−0.38
		1	34	116–378	0.12+/−0.21	0.06+/−0.09	0.00+/−0.00
Hematopoietic progenitor	Lin^−^Thy1.2^−^Sca-1^−/low^c-kit^+/low^	1.2–1.4×10^6^	3	29–31	62.9+/−18.7	82.3+/−4.4	16.9+/−8.2
		1.2–4.7×10^5^	3	27–29	37.9+/−21.3	51.8+/−27.5	14.4+/−7.4
		1.2–3.0×10^4^	4	21–29	4.6+/−5.3	3.8+/−2.4	10.8+/−4.4
	Myeloid progenitor	0.4–2.8×10^5^	7	24–39	4.2+/−5.2	3.6+/−4.1 d	12.8+/−10.7^b^
	CMP	0.3–1.0×10^5^	6	31–42	0.87+/−0.70	0.52+/−0.59	17.2+/−7.3 c
	GMP	0.9–1.3×10^5^	4	24–42	1.5+/−2.4	11.0+/−22.0	6.6+/−5.9
	CLP	0.2–3.3×10^5^	9	24–39	4.0+/−4.7	8.7+/−6.8^d^	0.67+/−1.00b,c

a P = 0.0095 by Mann-Whitney U test, Lin^−/low^CD45^+^ versus Lin^−/low^CD45^−.^

b P = 0.0021 by Mann-Whitney U test, Myeloid progenitor versus CLP.

c P = 0.0004 by Mann-Whitney U test, CMP versus CLP.

d P = 0.1142 by Mann-Whitney U test, Myeloid progenitor versus CLP.

* GFP^+^ cardiomyocytes were counted in 40 contiguous sections from apex of the heart per a mouse. Detailed information of histological analysis is described in Materials and Methods, Materials and Methods S1.

Abbreviations. BM: bone marrow, PB: peripheral blood, HSC: hematopoietic stem cell, LSK: Lin^−^Sca-1^+^c-Kit^+^, CMP: common myeloid progenitor, GMP: granulocyte/monocyte progenitor, CLP: common lymphoid progenitor.

### Transplantation of Purified BM HSC Fraction Resulted in the Efficient Production of Donor-derived Cardiomyocytes

**Figure 2:**
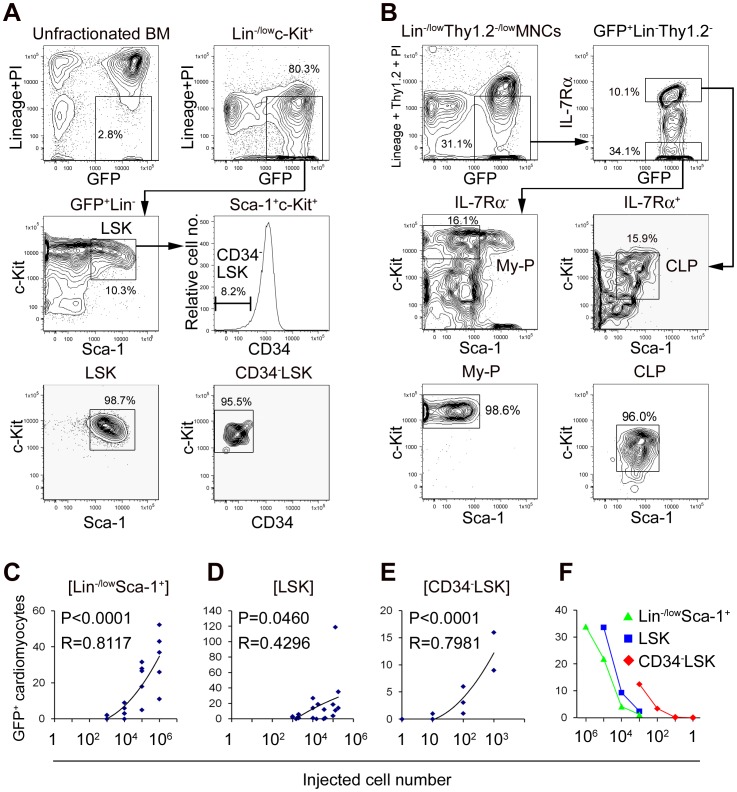
Transplantation of HSCs and hematopoietic progenitors. (A) LSKs or CD34^−^LSKs were purified by FACS from Lin^−/low^c-Kit^+^ BM fraction of GFP mice. GFP and Lineage+propidium iodide (PI) expression of unfractionated BM cells is shown as a control. (B) Total myeloid progenitors (My-P) and CLPs were purified by FACS from Lin^−/low^Thy1.2^−/low^MNCs of GFP mice. (C-E) Correlational analyses between injected cell numbers and the numbers of GFP^+^ cardiomyocytes per a recipient mouse in recipients transplanted with Lin^−/low^Sca-1^+^ cells (C), LSKs (D), and CD34^−^LSKs (E). (F) Comparison of the number of GFP^+^ cardiomyocytes at the same injected cell dose in Lin^−/low^Sca-1^+^ cells, LSKs, and CD34^−^LSKs recipients. The means of the number of GFP^+^ cardiomyocytes in recipients transplanted with each injected cell number are plotted. In the transplantation of LSKs, injected cell number of 1–1.8×10^5^ cells is plotted at 10^5^, that of 1–4×10^4^ cells is plotted at 10^4^, and that of 1–2×10^3^ cells is plotted at 10^3^. The greater cardiomyogenic ability existed in CD34^−^LSKs than LSKs, and in LSKs than Lin^−/low^Sca-1^+^ cells.

Next, we aimed to assess the cardiomyogenic ability of purified hematopoietic stem/progenitor population. We transplanted limiting numbers of GFP^+^Lin^−/low^Sca1^+^ cells including HSCs and hematopoietic progenitors, purified GFP^+^Lin^−^Sca-1^+^c-Kit^+^ cells (LSKs; Figure 2A) including HSCs and multipotent progenitors, and purified GFP^+^CD34^−^Lin^−^Sca-1^+^c-Kit^+^ cells (CD34^−^LSKs; Figure 2A) which contain HSCs that have long-term self-renewing potential. In all recipients transplanted with above three types of cells, GFP^+^ cells of myeloid lineage, B cell lineage, and T cell lineage were present in peripheral blood (PB) and BM (see Figure S2). The numbers of GFP^+^ cardiomyocytes significantly correlated with the injected cell dose in each group of the recipients transplanted with 10^3^–10^6^ Lin^−/low^Sca-1^+^ cells from GFP mice, 10^3^–1.8×10^5^ GFP^+^LSKs, and 1–10^3^ GFP^+^CD34^−^LSKs (P<0.05 in all groups; Figure 2C–E, Table 1). Furthermore, the incidence of GFP^+^ cardiomyocytes per injected cell dose was greatest in GFP^+^CD34^−^LSKs recipients followed by that in GFP^+^LSKs recipients, and the incidence in GFP^+^Lin^−/low^Sca-1^+^ recipients was the lowest (Figure 2F, Table 1). GFP^+^ cardiomyocytes were detected following transplantation of as few as 10 GFP^+^CD34^−^LSKs. We further confirmed contribution of self-renewing HSCs to the generation of cardiomyocytes by secondary and tertiary BM transplantation from GFP^+^LSK recipients (see Table S1). These findings indicate that BM cells with cardiomyogenic capacity are highly enriched within the CD34^−^LSK HSC population.

### Myeloid Lineage Cells are the Primary Intermediates for Generating Cardiomyocytes

Since the capacity of HSCs to convert directly into cardiomyocytes has yet to be determined [18]–[21], we examined the capacity of hematopoietic progenitors to act as relevant intermediates for cardiomyogenesis. At 4 weeks after transplantation of 1.2×10^4^–1.4×10^6^ fluorescence-activated cell sorting (FACS)-purified GFP^+^Lin^−^Thy1.2^−^Sca-1^−/low^c-Kit^+/low^ cells which mainly contain mixed myeloid and lymphoid progenitor population, GFP^+^ cardiomyocytes were identified in all recipients (n = 10; Table 1). This result indicates that transplanted HSCs give rise to cardiomyocytes via hematopoietic intermediates, at least partly.

To discriminate the hematopoietic lineage required in cardiomyogenesis, we then transplanted 0.4–2.8×10^5^ FACS-purified GFP^+^Lin^−^Thy1.2^−^interleukin-7 receptor α-chain (IL-7Rα)^−^Sca-1^−^c-Kit^+^ total myeloid progenitors (n = 7) and 0.2–3.3×10^5^ GFP^+^Lin^−^Thy1.2^−^IL-7Rα^+^Sca-1^low^c-Kit^low^ common lymphoid progenitors (CLPs; n = 9) sorted simultaneously from the same GFP^+^ BM cells (Figure 2B). At 3–6 weeks after transplantation, recipients of myeloid progenitors showed predominant GFP^+^ myeloid engraftment while recipients of CLPs showed predominant GFP^+^ lymphoid engraftment in PB (see Figure S2). Although recipients of myeloid progenitors and CLPs showed similar PB GFP^+^ engraftment (Table 1), significantly greater number of GFP^+^ cardiomyocytes were identified in recipients transplanted with myeloid progenitors than in those transplanted with CLPs (P = 0.0021; Table 1). We further confirmed the contribution of myeloid cells to the generation of cardiomyocytes by transplantation of GFP^+^Lin^−^IL-7Rα^−^Sca-1^−^c-Kit^+^ Fcγ receptor-II/III (FcγR)^low^CD34^+^ common myeloid progenitor (CMPs; 0.3–1.0×10^5^, n = 6; Table 1) and GFP^+^Lin^−^IL-7Rα^−^Sca-1^−^c-Kit^+^FcγR^high^CD34^+^ granulocyte/monocyte progenitors (GMPs; 0.9–1.3×10^5^, n = 4; Table 1). These findings suggest that myeloid derivatives are responsible for providing cardiomyocytes in recipients.

### Cell Fusion is the Major Mechanism Underlying the Generation of BM-derived Cardiomyocytes

**Figure 3:**
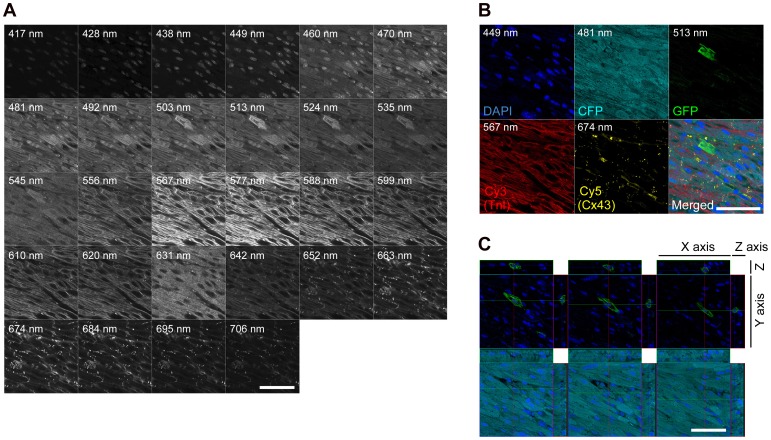
CFP expression in the GFP^+^ BM-derived cardiomyocyte of a CFP-transgenic recipient mouse. (A) Representative image of fluorescence detection from cardiac tissue of the CFP-transgenic recipient transplanted with GFP^+^ BM cells. Detected fluorescence images between 417 and 706 nm wavelength at 10–11 nm interval from a cardiac section stained with anti-TnI (Cy3), anti-Cx43 (Cy5), and DAPI are shown sequentially. (B) The expressions of DAPI, CFP, GFP, Cy3, and Cy5 in the cardiomyocyte shown in (A). Composition of GFP and CFP was examined simultaneously from each detected image between 449 and 663 nm wavelength (linear unmixing analysis). Detection wavebands used to visualize each fluorescence are described in each fluorescence image. The cardiomyocyte shown in (A) expressed donor-derived GFP, recipient-derived CFP, cardiac TnI, and Cx43. (C) Three dimensional analysis of the cardiomyocyte shown in (A) and (B) at the position of each nucleus. In every cross-section of three planes including nuclei, this cardiomyocyte expressed both GFP and CFP. Merged images (B and C) were obtained from the same confocal plane. Scale bars = 20 µm.

To explore the mechanism of BM-derived cardiomyocyte generation, BM cells of GFP mice were transplanted into cyan fluorescence protein (CFP)-transgenic newborn mice. At 4–22 weeks after transplantation of unfractionated BM cells, LSK, Lin^−^Thy1.2^−^Sca-1^−/low^c-Kit^+/low^, Lin^−/low^Sca-1^+^, and Lin^−/low^Sca-1^−^ cells from GFP mouse BM, 1–166 GFP^+^ cardiomyocytes per a mouse were detected in CFP recipients (n = 11). The incidence of GFP^+^ cardiomyocytes in CFP recipients was comparable to that in C57BL/6 recipients, suggesting that the cardiomyogenic event is not influenced by employing CFP-transgenic mice as recipients. The emitted fluorescence from recipient cardiac sections stained with cardiac TnI-cyanin-3 (Cy3), Cx43-cyanin-5 (Cy5), and 4′,6-diaimidino-2-phenylindole (DAPI) was detected at 10–11 nm interval from 417 nm to 749 nm wavelength using a laser-scanning confocal microscope (Figure 3A). The presence of cyan fluorescence in multiple points of each GFP^+^TnI^+^Cx43^+^ cardiomyocyte was examined by using linear unmixing analysis (see Materials and Methods S1). Irrespective of the donor cell type, all 21 GFP^+^ cardiomyocytes detected in the cardiac tissues of 8 recipients coexpressed CFP (Figure 3B and 3C, see Table S2), suggesting that BM-derived cardiomyocytes are generated through cell fusion between BM-derived cells and recipient-derived cardiomyocytes.

### Analysis of Cardiomyocyte-specific Genes in Mice Transplanted with Human Cord Blood HSCs

Finally, we aimed to clarify whether donor hematopoietic cells can change their cell fate through cell fusion with host cardiomyocytes. To this end, we set up xenogeneic transplantation by injecting human cord blood-derived CD34^+^CD38^−^ HSCs into immune-compromised mice (NOD/SCID/IL2rγ^null^ mice [23], NOD.Rag1^null^IL2rγ^null^ mice [24], and C57BL/6.Rag2^null^IL2rγ^null^NOD-Sirpa mice [25]). We extracted ribonucleic acid (RNA) from recipient cardiac tissues and performed quantitative real-time polymerase chain reaction (qRT-PCR) to detect human-specific cardiac transcription factors (Gata-4 [GATA4], Tbx 5 [TBX5], Nkx 2.5 [NKX2–5], and Mesp 1 [MESP1]) [26] and cardiomyocyte structural genes (α-myosin heavy chain 6 [MYH6] and cardiac troponin C [TNNC1], connexin 43 [GJA1], and ryanodine receptor 2 [RYR2]). Of cardiac transcription factors, Gata-4, Tbx 5, and Mesp 1 were not expressed in any of the examined 14 recipients. Although Nkx 2.5 was expressed in 2 of 14 recipients, the expression of Nkx 2.5 was not specific to human cardiomyocytes since it was expressed in human cord blood [27] (see Table S3). Of cardiomyocyte structural genes, human myosin heavy chain 6 and troponin C were expressed in 1 of 14 and 9 of 14 recipients, respectively. Specificity of these probes to human cardiomyocytes was confirmed by the positive reaction to human heart and the negative reaction to mouse heart and human cord blood. By contrast, ryanodine receptor 2 was not detected in any cardiac specimens derived from human HSC-engrafted recipients. Although connexin 43 was expressed in all of the recipients, the expression of connexin 43 was not specific to human cardiomyocytes since it was expressed in human cord blood consistent with previous reports showing connexin expression on several hematopoietic cells [28] (see Table S3). Although the frequency of cell fusion between donor hematopoietic cells and host cardiomyocytes is extremely low, we detected the cardiomyocyte-specific expression of the human cardiomyocyte structural gene, cardiac troponin C, but not that of other human cardiomyocyte structural genes such as ryanodine receptor 2 or human cardiac transcription factors in the cardiac tissue of recipient mice.

## Discussion

We have obtained three findings by tracing the fate of genetically marked BM cells *in vivo*. First, HSCs generate cardiomyocytes along with hematopoietic reconstitution. Second, myeloid lineage cells are primary intermediates for cardiomyocyte generation. Third, cell fusion with recipient-derived cardiomyocytes is required for the generation of BM-derived cardiomyocytes. In this study, transplantation of cells derived from GFP-transgenic mouse into CFP-transgenic recipients enabled us to determine the mechanism for the generation of donor marker-positive cardiomyocytes. Moreover, confocal imaging and analyses for the separation of these two fluorescences led us to address the long-term question as to the issue of cell fusion or transdifferentiation.

Transplantation of purified HSCs to newborn syngeneic mice with cardiac injury resulted in the most frequent appearance of donor-marker^+^ cardiomyocytes in cardiac tissues. The results suggest the two possibilities, direct contribution of HSCs to cell fusion with cardiomyocytes and cell fusion between myeloid progeny and cardiomyocytes. Previous reports suggest that purified HSCs do not generate cardiomyocytes when directly transplanted into myocardium [10], [20], [21], but can generate cardiomyocytes following BM transplantation at a low frequency [4], [10], [12]. We have shown that cell fusion between BM-derived myeloid cells and host-derived cardiomyocytes results in the generation of donor-marker^+^ cardiomyocytes. The frequency of BM-derived cardiomyocytes in our study (∼0.04%, see Materials and Methods S1) and other rodent studies (0.001–0.02%) [4], [10], [20] are similar to those of host marker-expressing cardiomyocytes in human cardiac transplantation (0.04–0.16%) [29], [30], which may be the frequency of fusion between circulating myeloid cells from BM and cardiomyocytes in mammals in general.

Cells of myeloid lineage have been shown to contribute to skeletal myocytes [31], [32], hepatocytes [33], [34], or vascular endothelium [35] *in vivo*. We report here contribution of BM-derived myeloid progenitors to the generation of cardiomyocytes. Actually, several plasma membrane molecules related with cell fusion were up-regulated in myeloid lineage cells than lymphoid lineage cells confirmed by qRT-PCR (see Figure S3). Expression levels of integrin beta 1 [Itgb1] or signal-regulatory protein alpha [Sirpa] in CMP were 1.73 or 1.82 fold higher than those in CLP, respectively (n = 3). Of the cells derived from GMPs, monocytic cells are likely candidates as fusion partners since they have a physiological capacity for cell fusion [36]. Based on the finding that CD11b^low^ cells, rather than CD11b^high^ cells, within Gr-1^−/low^c-Kit^−^ BM fraction generated cardiomyocytes (unpublished observations), CD11b^low^ immature monocytes may be the population contributing to the appearance of donor-marker^+^ cardiomyocytes similar to the previous report describing the regeneration of skeletal muscle [32].

The number of donor-derived cells in recipient BM was very low following the transplantation of Lin^−/low^CD45^−^ cells in our study (Table 1), consistent with previous studies [37], [38]. Mesenchymal stem cells in the BM are considered to be relatively resistant to irradiation compared with HSCs [37], [38]. Therefore, the lack of donor-derived cardiomyocytes following systemic infusion of Lin^−/low^CD45^−^ cells into sublethally irradiated mice may be due to inefficient engraftment of injected MSCs in the recipient BM. Intra-BM injection of 10^5^ GFP-labeled clonally purified MSCs (CMG cells) resulted in the generation of donor-marker^+^ cardiomyocytes [11]. Our results, together with the report showing little ability of MSCs to give rise to cardiomyocytes following intravenous or intramyocardial transplantation [8], imply that administration route and cell preparation need to be extensively assessed for cell therapy using MSCs. We examined whether BM-derived mesenchymal cells gain a cardiomyocyte phenotype through differentiation or cell fusion with a coculture experiment using GFP-expressing BM mesenchymal cells and adult cardiomyocytes. In the experiment, one GFP^+^ cell expressing cardiomyocyte-specific antigens was detected (unpublished observations). However, the function of the cardiac-maker positive cell as a cardiomyocyte is unknown. A previous study demonstrated that mesenchymal stromal cell-derived cells acquiring cardiomyocyte phenotypic characteristics do not exhibit sufficient electrical property [39]. Additionally, improvement of cardiac function via mesenchymal stem/stromal cell transplantation is thought to be mediated by paracrine mechanism in addition to the direct contribution of cardiac marker-positive mesenchymal stem/stromal cells in themselves as contractile cardiomyocytes [40], [41]. Recent report suggests that mesenchymal stromal cells recruit monocytes/macrophages secreting IL-10 or TGF-β [41]. Macrophages contributing to anti-inflammatory status could be one of the determinants for tissue regeneration in a mouse model for myocardial infarction [41]. Therefore, whether HSCs and MSCs act synergistically to regenerate cardiac tissue or to inhibit apoptosis in damaged cardiac tissue should be evaluated by using a large animal model in the future.

To establish regenerative medicine for heart failure patients, long-term improvement in cardiac function is important. Self-renewing cardiac-resident stem/progenitor cells [42], [43] could maintain a pool for cardiomyocyte turnover [44], [45]. BM could be one of the reservoirs that can give rise to cardiac-resident stem cells [46] and cells of multiple lineages [47]. Indeed, cardiomyocyte renewal in adult mammals is demonstrated under the physiological condition [48] or after injury [1]. Therefore, it should be demonstrated in the future to what degree BM-derived stem/progenitor cells and cardiac stem/progenitor cells contribute to turnover and regeneration of cardiomyocytes in the physiological or pathological conditions and whether BM-derived stem/progenitor cells can generate cardiac stem/progenitor cells.

Finally, we have demonstrated the contribution of myeloid lineage cells to cardiomyocytes through cell fusion. Recent reports suggest that stem cell fusion with the cardiomyocyte could add contractile function to the injured heart [49], and BM cell transplantation can restore impaired cardiac function [7]. Identification of the specific cell type and factors involved in cell fusion may lead to the enhancement of therapeutic effects of ongoing clinical cell therapy. In our qPCR analysis, two out of 6 donor cardiomyocyte-specific genes were detected in recipient cardiac tissues. Considering the low frequency of cell fusion, future *in vitro* and *in vivo* experiments might be required to determine as to how far reprogramming process occurs following cell fusion. Altogether, our findings suggest that myeloid progenitors may potentially serve as a readily available source of effecter cells for targeted cellular therapy of cardiac disorders through cell fusion.

## Materials and Methods

### Mice

C57BL/6 mice transgenically expressing enhanced GFP driven by the cytomegalovirus (CMV) enhancer/chicken β-actin promoter were kindly provided by Dr. M. Okabe (Osaka University). C57BL/6 mice transgenically expressing enhanced CFP driven by the CMV enhancer/chicken β-actin promoter, NOD/SCID/IL2rγ^null^ mice, and NOD.Rag1^null^IL2rγ^null^ mice were obtained from the Jackson Laboratory (Bar Harbor, ME). C57BL/6.Rag2^null^IL2rγ^null^NOD-Sirpa mice were generated in our laboratory. All mice were bred and maintained under defined flora. Experiments were performed according to the guidelines approved by the Institutional Animal Committee of Kyushu University (Approval ID: 18–68). PB sampling from recipient mice was performed under deep inhalation anesthesia. BM and heart were dissected out from donor/recipient mice sacrificed by cervical dislocation under deep inhalation anesthesia.

### Purification of Donor Cells

BM cells were harvested from GFP-transgenic mice at 8–12 weeks of age by flushing femurs and tibiae. MNCs were isolated by gradient centrifugation. Rat anti-mouse lineage antigen (BD Pharmingen, Caltag) and sheep anti-rat IgG antibody-conjugated immunomagnetic beads (Dynal) were used to deplete mature hematopoietic cells from MNCs. For separation of Lin^−/low^CD45^+^ cells and Lin^−/low^CD45^−^ cells and enrichment of Lin^−/low^Sca-1^+^ cells or Lin^−/low^c-Kit^+^ cells by magnetic cell sorting, immunomagnetic microbeads (Miltenyi Biotec) were used. For purification of each BM stem/progenitor fraction by FACS, enriched BM cells were labeled by fluorescence-conjugated antibodies (see Materials and Methods S1). Umbilical cord blood cells were collected during normal full-term delivers after obtained written informed consent (provided by Kyushu Block Red Cross Blood Center, Japan Red Cross Society). Experiments using cord blood were performed according to the guidelines approved by the Institutional Committee of Kyushu University (Approval ID: 17–114). MNCs were separated by Ficoll-Hypaque density-gradient centrifugation. Lineage-depleted cord blood cells were obtained magnetically (Miltenyi Biotec). Sorting of CD34^+^CD38^−^ subfractions was accomplished by staining lineage-depleted cord blood cells with fluorescein isothiocyanate (FITC)-conjugated anti-CD34, and phycoerythrin (PE)-conjugated anti-CD38 (BD Biosciences). Cell sorting was performed on a FACSAria (BD).

### Transplantation

Unfractionated or purified BM cells from GFP mice were injected intravenously into C57BL/6 mice or CFP-transgenic mice within 48 hours of birth after 560 cGy irradiation. Left ventricular wall injury was induced by puncturing newborn recipient heart with a 29 G needle following transplantation of donor BM cells. Five to ten thousand Lin^−^CD34^+^CD38^−^ cells from human cord blood were transplanted into immune-compromised mice after irradiation.

### Flow Cytometric Analysis of Recipient PB and BM

PB was collected from retro-orbital plexus of recipients, and BM cells were harvested from femurs and tibiae. The percentage of GFP^+^ cells in PB and BM of recipients was examined by flow cytometric analysis using FACSCalibur (BD). PB and BM of recipients were labeled with each biotin-conjugated anti-mouse lineage antigen (CD3e, CD11b, B220, Gr-1, and TER-119) antibody, subsequently labeled with Cy3-conjugated streptavidin (Jackson ImmunoResearch) to recognize myeloid cells (CD11b, Gr-1), T lymphocytes (CD3e), and B lymphocytes (B220) by flow cytometric analysis. FACS lysing solution (BD) was used to lyse red blood cells following antibody labeling.

### Histological Analysis

Recipient hearts were removed and perfused with PBS to eliminate blood cells immediately after they were sacrificed by cervical dislocation. For immunofluorescence staining, recipient hearts were sectioned into 100-µm slices from apex to base with a vibratome (DTK-1000, D.S.K.) following fixation in 4% paraformaldehyde and dehydration in 70% ethanol. Vibratome-sectioned cardiac slices were stained with primary antibodies to CD45 (DakoCytomation), CD11b (BD Pharmingen), vimentin (Sigma), cardiac troponin I (TnI; Santa Cruz Biotechnology), and connexin43 (Cx43; Chemicon). Secondary antibodies conjugated with Cy3 or Cy5 (Jackson ImmunoResearch) were used to visualize primary antibodies. Forty contiguous cardiac sections per a recipient mouse were carefully examined for the presence of GFP^+^ cardiomyocytes by laser-scanning confocal microscopy (LSM-GB200, Olympus). In the transplantation of GFP^+^ BM cells into CFP-transgenic mice, linear unmixing analysis was employed to distinguish GFP^+^ donor-derived cells, GFP^+^CFP^+^ fused cells, and CFP^+^ recipient-derived cells as described previously [12] (LSM510 META, Carl Zeiss, see Materials and Methods S1).

### Quantitative Real-time Polymerase Chain Reaction

Total RNA was extracted from recipient heart, non-recipient mouse heart, human cord blood, and 10^4^ cells each of sorted mouse hematopoietic cells. Reverse transcription was performed using SuperScript II or SuperScript III (Invitrogen) according to the manufacturer’s instructions. For human-specific cardiac transcription factors and cardiomyocyte structural genes, qRT-PCR was performed on the synthesized complementary deoxyribonucleic acid (cDNA) with Platinum Quantitative PCR SuperMix (Invitrogen) on LightCycler 480 (Roche) to determine cycle thresholds (C_T_) of amplification. Human heart QUICK-Clone cDNA (Clontech) was used as a positive control. Human glyceraldehyde-3-phosphate dehydrogenase (GAPDH) was used as the internal control. For mouse adhesion molecules, qRT-PCR was performed on Applied Biosystems 7500 real-time PCR system with TaqMan^(R)^ primer-probe sets provided from Applied Biosystems. 18 s rRNA was used as the internal control. Sequences of dual-labeled fluorogenic probes and gene-specific primers (Sigma-Aldrich), and product ID of TaqMan^(R)^ Gene Expression Assay (Applied Biosystems) are listed on Table S4. For initial normalization to the housekeeping gene, the difference in C_T_ value (ΔC_T_ = [C_T_ gene of interest] – [C_T_ human GAPDH or 18 s rRNA]) was determined on each sample. Relative expression levels of each mRNA were calculated as the ratio to levels of human heart or mouse CLP cDNA using the comparative C_T_ (2^−ΔΔCt^) method [50].

### Statistical Analysis

Results are presented as mean +/− standard deviation (S.D.). Probability values were calculated by using the Mann-Whitney U test (non-parametric independent two-group comparison) or the Pearson’s correlation coefficient test. A probability value of less than 0.05 was considered to be statistically significant.

## Supporting Information

Figure S1 Differentiation capacities of Lin^−/low^CD45^−^ cells. (A) CD45 expression of Lin^−/low^MNCs, separated Lin^−/low^CD45^+^ cells, and separated Lin^−/low^CD45^−^ cells. (B) Bright-field and fluorescence image of GFP^+^Lin^−/low^CD45^−^ cells following culture. Polygonal or spindle-shaped adherent GFP^+^ cells were recognized as mesenchymal cells. (C) *In vitro* differentiation of Lin^−/low^CD45^−^ cells into osteoblasts (left) or adipocytes (right) by induction using differentiation media for each. Differentiation into osteoblasts or adipocytes was confirmed by alkaline phosphatase staining (left; red) or Oil red O staining (right; lipid vacuoles are stained in red), respectively. (D) Bone section of recipients transplanted with GFP^+^Lin^−/low^CD45^−^ cells stained with anti-GFP (red, Cy3), anti-CD45 (yellow, Cy5), and DAPI (blue). Bright-field image is shown at leftmost. GFP^+^CD45^−^ cells were present within bone cortex (white arrows). These results suggest that separated Lin^−/low^CD45^−^ cells contain MSCs that can differentiate into multiple mesenchymal lineages. Merged images were obtained from the same confocal plane. Scale bars = 20 µm.
(TIF)

Figure S2 Flow cytometric analysis of recipient hematopoietic tissues. Representative image of flow cytometric analysis of recipient PB and BM transplanted with FACS-purified LSKs, and recipient PB transplanted with total myeloid progenitors (My-P) or CLPs. In LSK recipients, donor-derived GFP^+^ myeloid cell lineage (Gr-1^+^ or CD11b^+^), B cell lineage (B220^+^), and T cell lineage (CD3e^+^) were confirmed. Donor-derived myeloid cell lineage (Gr-1^+^ or CD11b^+^) was predominantly present in recipients transplanted with total myeloid progenitors, and donor-derived B/T cell lineage (B220^+^ or CD3e^+^) was predominantly present in recipients transplanted with CLPs.
(TIF)

Figure S3 Relative cDNA expression of adhesion molecules in myeloid and lymphoid lineages. The averages of relative cDNA expressions from three qRT-PCR experiments are indicated by histogram with positive standard deviation. Expression levels of Itgb1 and Sirpa in CMP were higher than those in CLP. Expression levels of Itgb1, Sirpa, and Adam9 in myeloid derivatives (neutrophil, monocyte) were higher than those in lymphoid derivatives (T lymphocyte, B lymphocyte). In the four adhesion molecules examined (Itgb1, Sirpa, Adam9, and Adam12), Adam12 was not detected in any of the samples. Gene names corresponding to each gene symbol, probes and primers information are described in Table S4.
(TIF)

Table S1 The number of donor-derived cardiomyocytes after serial transplantation.
(XLS)

Table S2 CFP expression of donor-derived GFP^+^ cardiomyocytes in CFP recipient mice.
(XLS)

Table S3 Relative cDNA expression to human heart.
(XLS)

Table S4 Probes and primers for quantitative real-time polymerase chain reaction (qRT-PCR).
(XLS)

Materials and Methods S1 Detailed information on purification of donor BM cells, cell culture and differentiation assay of Lin^−/low^CD45^−^ cells, and histological analysis.
(DOC)

## References

[1] Hsieh PC, Segers VF, Davis ME, MacGillivray C, Gannon J, et al (2007) Evidence from a genetic fate-mapping study that stem cells refresh adult mammalian cardiomyocytes after injury. Nat Med 13: 970–974. 17660827 10.1038/nm1618 PMC2754571

[2] Dimmeler S, Zeiher AM, Schneider MD (2005) Unchain my heart: the scientific foundations of cardiac repair. J Clin Invest 115: 572–583. 15765139 10.1172/JCI24283 PMC1052009

[3] Orlic D, Kajstura J, Chimenti S, Jakoniuk I, Anderson SM, et al (2001) Bone marrow cells regenerate infarcted myocardium. Nature 410: 701–705. 11287958 10.1038/35070587

[4] Jackson KA, Majka SM, Wang H, Pocius J, Hartley CJ, et al (2001) Regeneration of ischemic cardiac muscle and vascular endothelium by adult stem cells. J Clin Invest 107: 1395–1402. 11390421 10.1172/JCI12150 PMC209322

[5] Schächinger V, Erbs S, Elsässer A, Haberbosch W, Hambrecht R, et al (2006) Intracoronary bone marrow-derived progenitor cells in acute myocardial infarction. N Engl J Med 355: 1210–1221. 16990384 10.1056/NEJMoa060186

[6] Meyer GP, Wollert KC, Lotz J, Steffens J, Lippolt P, et al (2006) Intracoronary bone marrow cell transfer after myocardial infarction: eighteen months' follow-up data from the randomized, controlled BOOST (BOne marrOw transfer to enhance ST-elevation infarct regeneration) trial. Circulation 113: 1287–1294. 16520413 10.1161/CIRCULATIONAHA.105.575118

[7] Jeevanantham V, Butler M, Saad A, Abdel-Latif A, Zuba-Surma EK, et al (2012) Adult bone marrow cell therapy improves survival and induces long-term improvement in cardiac parameters: a systematic review and meta-analysis. Circulation 126: 551–568. 22730444 10.1161/CIRCULATIONAHA.111.086074 PMC4282649

[8] Breitbach M, Bostani T, Roell W, Xia Y, Dewald O, et al (2007) Potential risks of bone marrow cell transplantation into infarcted hearts. Blood 110: 1362–1369. 17483296 10.1182/blood-2006-12-063412

[9] Koide Y, Morikawa S, Mabuchi Y, Muguruma Y, Hiratsu E, et al (2007) Two distinct stem cell lineages in murine bone marrow. Stem Cells 25: 1213–1221. 17218403 10.1634/stemcells.2006-0325

[10] Nygren JM, Jovinge S, Breitbach M, Säwén P, Röll W, et al (2004) Bone marrow-derived hematopoietic cells generate cardiomyocytes at a low frequency through cell fusion, but not transdifferentiation. Nat Med 10: 494–501. 15107841 10.1038/nm1040

[11] Kawada H, Fujita J, Kinjo K, Matsuzaki Y, Tsuma M, et al (2004) Nonhematopoietic mesenchymal stem cells can be mobilized and differentiate into cardiomyocytes after myocardial infarction. Blood 104: 3581–3587. 15297308 10.1182/blood-2004-04-1488

[12] Ishikawa F, Shimazu H, Shultz LD, Fukata M, Nakamura R, et al (2006) Purified human hematopoietic stem cells contribute to the generation of cardiomyocytes through cell fusion. FASEB J 20: 950–952. 16585061 10.1096/fj.05-4863fje

[13] Toma C, Pittenger MF, Cahill KS, Byrne BJ, Kessler PD (2002) Human mesenchymal stem cells differentiate to a cardiomyocyte phenotype in the adult murine heart. Circulation 105: 93–98. 11772882 10.1161/hc0102.101442

[14] Terada N, Hamazaki T, Oka M, Hoki M, Mastalerz DM, et al (2002) Bone marrow cells adopt the phenotype of other cells by spontaneous cell fusion. Nature 416: 542–545. 11932747 10.1038/nature730

[15] Alvarez-Dolado M, Pardal R, Garcia-Verdugo JM, Fike JR, Lee HO, et al (2003) Fusion of bone-marrow-derived cells with Purkinje neurons, cardiomyocytes and hepatocytes. Nature 425: 968–973. 14555960 10.1038/nature02069

[16] Weimann JM, Johansson CB, Trejo A, Blau HM (2003) Stable reprogrammed heterokaryons form spontaneously in Purkinje neurons after bone marrow transplant. Nat Cell Biol 5: 959–966. 14562057 10.1038/ncb1053

[17] Herzog EL, Van Arnam J, Hu B, Zhang J, Chen Q, et al (2007) Lung-specific nuclear reprogramming is accompanied by heterokaryon formation and Y chromosome loss following bone marrow transplantation and secondary inflammation. FASEB J 21: 2592–2601. 17449722 10.1096/fj.06-7861com

[18] Jang YY, Collector MI, Baylin SB, Diehl AM, Sharkis SJ (2004) Hematopoietic stem cells convert into liver cells within days without fusion. Nat Cell Biol 6: 532–539. 15133469 10.1038/ncb1132

[19] Harris RG, Herzog EL, Bruscia EM, Grove JE, Van Arnam JS, et al (2004) Lack of a fusion requirement for development of bone marrow-derived epithelia. Science 305: 90–93. 15232107 10.1126/science.1098925

[20] Murry CE, Soonpaa MH, Reinecke H, Nakajima H, Nakajima HO, et al (2004) Haematopoietic stem cells do not transdifferentiate into cardiac myocytes in myocardial infarcts. Nature 428: 664–668. 15034593 10.1038/nature02446

[21] Balsam LB, Wagers AJ, Christensen JL, Kofidis T, Weissman IL, et al (2004) Haematopoietic stem cells adopt mature haematopoietic fates in ischaemic myocardium. Nature 428: 668–673. 15034594 10.1038/nature02460

[22] Conboy IM, Conboy MJ, Wagers AJ, Girma ER, Weissman IL, et al (2005) Rejuvenation of aged progenitor cells by exposure to a young systemic environment. Nature 433: 760–764. 15716955 10.1038/nature03260

[23] Ishikawa F, Yasukawa M, Lyons B, Yoshida S, Miyamoto T, et al (2005) Development of functional human blood and immune systems in NOD/SCID/IL2 receptor {gamma} chain(null) mice. Blood 106: 1565–1573. 15920010 10.1182/blood-2005-02-0516 PMC1895228

[24] Pearson T, Shultz LD, Miller D, King M, Laning J, et al (2008) Non-obese diabetic-recombination activating gene-1 (NOD-Rag1 null) interleukin (IL)-2 receptor common gamma chain (IL2 r gamma null) null mice: a radioresistant model for human lymphohaematopoietic engraftment. Clin Exp Immunol 154: 270–284. 18785974 10.1111/j.1365-2249.2008.03753.x PMC2612717

[25] Yamauchi T, Takenaka K, Urata S, Shima T, Kikushige Y, et al (2013) Polymorphic Sirpa is the genetic determinant for NOD-based mouse lines to achieve efficient human cell engraftment. Blood 121: 1316–1325. 23293079 10.1182/blood-2012-06-440354

[26] Bruneau BG (2002) Transcriptional regulation of vertebrate cardiac morphogenesis. Circ Res 90: 509–519. 11909814 10.1161/01.res.0000013072.51957.b7

[27] Kucia M, Halasa M, Wysoczynski M, Baskiewicz-Masiuk M, Moldenhawer S, et al (2007) Morphological and molecular characterization of novel population of CXCR4+ SSEA-4+ Oct-4+ very small embryonic-like cells purified from human cord blood- preliminary report. Leukemia 21: 297–303. 17136117 10.1038/sj.leu.2404470

[28] Neijssen J, Pang B, Neefjes J (2007) Gap junction-mediated intercellular communication in the immune system. Prog Biophys Mol Biol 94: 207–218. 17467043 10.1016/j.pbiomolbio.2007.03.008

[29] Laflamme MA, Myerson D, Saffitz JE, Murry CE (2002) Evidence for cardiomyocyte repopulation by extracardiac progenitors in transplanted human hearts. Circ Res 90: 634–640. 11934829 10.1161/01.res.0000014822.62629.eb

[30] Müller P, Pfeiffer P, Koglin J, Schäfers HJ, Seeland U, et al (2002) Cardiomyocytes of noncardiac origin in myocardial biopsies of human transplanted hearts. Circulation 106: 31–35. 12093766 10.1161/01.cir.0000022405.68464.ca

[31] Camargo FD, Green R, Capetanaki Y, Jackson KA, Goodell MA (2003) Single hematopoietic stem cells generate skeletal muscle through myeloid intermediates. Nat Med 9: 1520–1527. 14625546 10.1038/nm963

[32] Doyonnas R, LaBarge MA, Sacco A, Charlton C, Blau HM (2004) Hematopoietic contribution to skeletal muscle regeneration by myelomonocytic precursors. Proc Natl Acad Sci U S A 101: 13507–13512. 15353585 10.1073/pnas.0405361101 PMC518787

[33] Camargo FD, Finegold M, Goodell MA (2004) Hematopoietic myelomonocytic cells are the major source of hepatocyte fusion partners. J Clin Invest 113: 1266–1270. 15124017 10.1172/JCI21301 PMC398434

[34] Willenbring H, Bailey AS, Foster M, Akkari Y, Dorrell C, et al (2004) Myelomonocytic cells are sufficient for therapeutic cell fusion in liver. Nat Med 10: 744–748. 15195088 10.1038/nm1062

[35] Bailey AS, Willenbring H, Jiang S, Anderson DA, Schroeder DA, et al (2006) Myeloid lineage progenitors give rise to vascular endothelium. Proc Natl Acad Sci U S A 103: 13156–13161. 16920790 10.1073/pnas.0604203103 PMC1559769

[36] Helming L, Gordon S (2007) The molecular basis of macrophage fusion. Immunobiology 212: 785–793. 18086379 10.1016/j.imbio.2007.09.012

[37] Simmons PJ, Przepiorka D, Thomas ED, Torok-Storb B (1987) Host origin of marrow stromal cells following allogeneic bone marrow transplantation. Nature 328: 429–432. 2886914 10.1038/328429a0

[38] Laver J, Jhanwar SC, O’Reilly RJ, Castro-Malaspina H (1987) Host origin of the human hematopoietic microenvironment following allogeneic bone marrow transplantation. Blood 70: 1966–1968. 3315046

[39] Rose RA, Jiang H, Wang X, Helke S, Tsoporis JN, et al (2008) Bone marrow-derived mesenchymal stromal cells express cardiac-specific markers, retain the stromal phenotype, and do not become functional cardiomyocytes in vitro. Stem Cells 26: 2884–2892. 18687994 10.1634/stemcells.2008-0329

[40] Noiseux N, Gnecchi M, Lopez-Ilasaca M, Zhang L, Solomon SD, et al (2006) Mesenchymal stem cells overexpressing Akt dramatically repair infarcted myocardium and improve cardiac function despite infrequent cellular fusion or differentiation. Mol Ther 14: 840–850. 16965940 10.1016/j.ymthe.2006.05.016

[41] Dayan V, Yannarelli G, Billia F, Filomeno P, Wang XH, et al (2011) Mesenchymal stromal cells mediate a switch to alternatively activated monocytes/macrophages after acute myocardial infarction. Basic Res Cardiol 106: 1299–1310. 21901289 10.1007/s00395-011-0221-9

[42] Ferreira-Martins J, Ogórek B, Cappetta D, Matsuda A, Signore S, et al (2012) Cardiomyogenesis in the developing heart is regulated by c-kit-positive cardiac stem cells. Circ Res 110: 701–715. 22275487 10.1161/CIRCRESAHA.111.259507 PMC3292662

[43] Bu L, Jiang X, Martin-Puig S, Caron L, Zhu S, et al (2009) Human ISL1 heart progenitors generate diverse multipotent cardiovascular cell lineages. Nature 460: 113–117. 19571884 10.1038/nature08191

[44] Quaini F, Urbanek K, Beltrami AP, Finato N, Beltrami CA, et al (2002) Chimerism of the transplanted heart. N Engl J Med 346: 5–15. 11777997 10.1056/NEJMoa012081

[45] Genead R, Danielsson C, Andersson AB, Corbascio M, Franco-Cereceda A, et al (2010) Islet-1 cells are cardiac progenitors present during the entire lifespan: from the embryonic stage to adulthood. Stem Cells Dev 19: 1601–1615. 20109033 10.1089/scd.2009.0483

[46] Barile L, Cerisoli F, Frati G, Gaetani R, Chimenti I, et al (2011) Bone marrow-derived cells can acquire cardiac stem cells properties in damaged heart. J Cell Mol Med 15: 63–71. 19912439 10.1111/j.1582-4934.2009.00968.x PMC3822494

[47] Kajstura J, Rota M, Whang B, Cascapera S, Hosoda T, et al (2005) Bone marrow cells differentiate in cardiac cell lineages after infarction independently of cell fusion. Circ Res 96: 127–137. 15569828 10.1161/01.RES.0000151843.79801.60

[48] Bergmann O, Bhardwaj RD, Bernard S, Zdunek S, Barnabé-Heider F, et al (2009) Evidence for cardiomyocyte renewal in humans. Science 324: 98–102. 19342590 10.1126/science.1164680 PMC2991140

[49] Metzele R, Alt C, Bai X, Yan Y, Zhang Z, et al (2011) Human adipose tissue-derived stem cells exhibit proliferation potential and spontaneous rhythmic contraction after fusion with neonatal rat cardiomyocytes. FASEB J 25: 830–839. 21059751 10.1096/fj.09-153221 PMC3470720

[50] Schmittgen TD, Livak KJ (2008) Analyzing real-time PCR data by the comparative C(T) method. Nat Protoc 3: 1101–1108. 18546601 10.1038/nprot.2008.73

