# Supplementary figures and images for: Contribution of Bone Marrow-Derived Hematopoietic Stem/Progenitor Cells to the Generation of Donor-Marker+ Cardiomyocytes In Vivo
Source: PLoS One. 2013 May 7;8(5):e62506. doi: 10.1371/journal.pone.0062506 (PMC3647070; doi:10.1371/journal.pone.0062506)

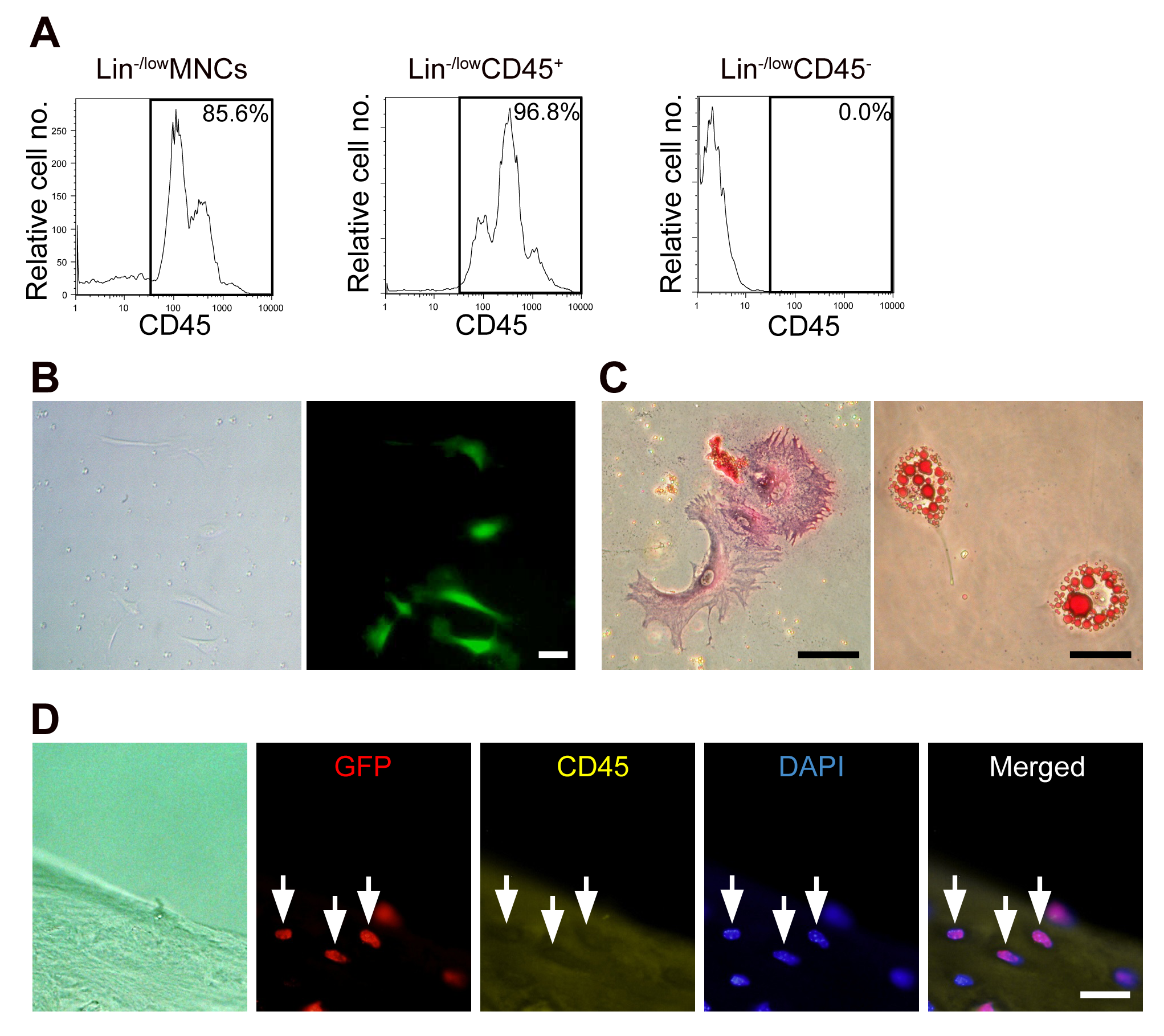

Supplement: Figure S1 — Differentiation capacities of Lin−/lowCD45− cells. (A) CD45 expression of Lin−/lowMNCs, separated Lin−/lowCD45+ cells, and separated Lin−/lowCD45− cells. (B) Bright-field and fluorescence image of GFP+Lin−/lowCD45− cells following culture. Polygonal or spindle-shaped adherent GFP+ cells were recognized as mesenchymal cells. (C) In vitro differentiation of Lin−/lowCD45− cells into osteoblasts (left) or adipocytes (right) by induction using differentiation media for each. Differentiation into osteoblasts or adipocytes was confirmed by alkaline phosphatase staining (left; red) or Oil red O staining (right; lipid vacuoles are stained in red), respectively. (D) Bone section of recipients transplanted with GFP+Lin−/lowCD45− cells stained with anti-GFP (red, Cy3), anti-CD45 (yellow, Cy5), and DAPI (blue). Bright-field image is shown at leftmost. GFP+CD45− cells were present within bone cortex (white arrows). These results suggest that separated Lin−/lowCD45− cells contain MSCs that can differentiate into multiple mesenchymal lineages. Merged images were obtained from the same confocal plane. Scale bars = 20 µm. (TIF) [file pone.0062506.s001.tif]

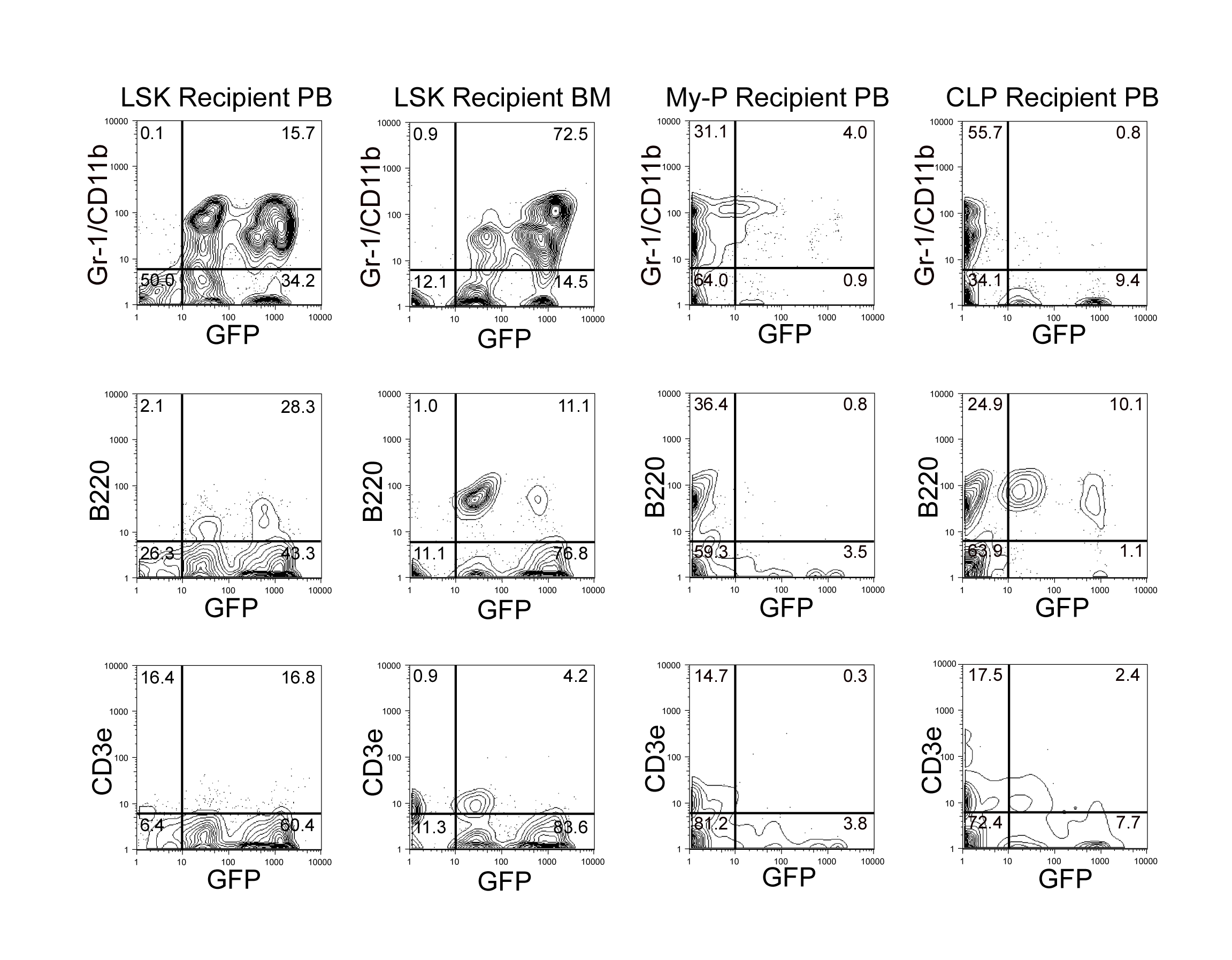

Supplement: Figure S2 — Flow cytometric analysis of recipient hematopoietic tissues. Representative image of flow cytometric analysis of recipient PB and BM transplanted with FACS-purified LSKs, and recipient PB transplanted with total myeloid progenitors (My-P) or CLPs. In LSK recipients, donor-derived GFP+ myeloid cell lineage (Gr-1+ or CD11b+), B cell lineage (B220+), and T cell lineage (CD3e+) were confirmed. Donor-derived myeloid cell lineage (Gr-1+ or CD11b+) was predominantly present in recipients transplanted with total myeloid progenitors, and donor-derived B/T cell lineage (B220+ or CD3e+) was predominantly present in recipients transplanted with CLPs. (TIF) [file pone.0062506.s002.tif]

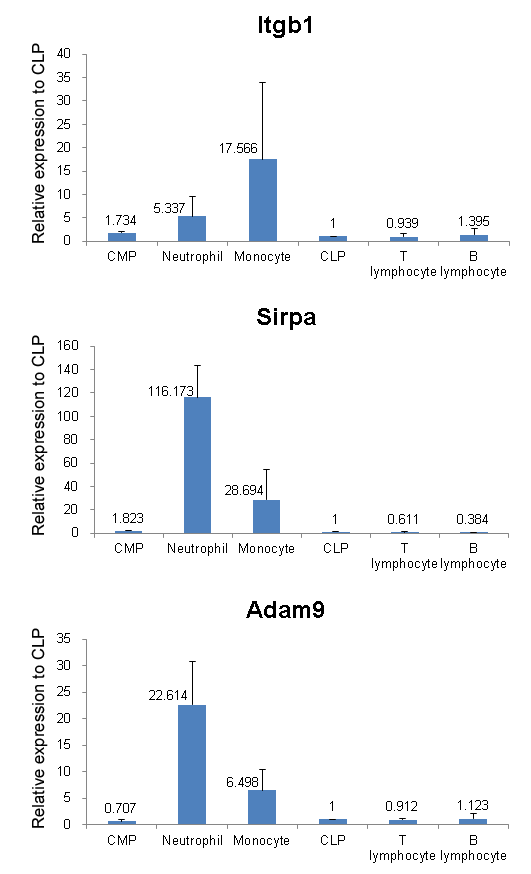

Supplement: Figure S3 — Relative cDNA expression of adhesion molecules in myeloid and lymphoid lineages. The averages of relative cDNA expressions from three qRT-PCR experiments are indicated by histogram with positive standard deviation. Expression levels of Itgb1 and Sirpa in CMP were higher than those in CLP. Expression levels of Itgb1, Sirpa, and Adam9 in myeloid derivatives (neutrophil, monocyte) were higher than those in lymphoid derivatives (T lymphocyte, B lymphocyte). In the four adhesion molecules examined (Itgb1, Sirpa, Adam9, and Adam12), Adam12 was not detected in any of the samples. Gene names corresponding to each gene symbol, probes and primers information are described in Table S4. (TIF) [file pone.0062506.s003.tif]
