# Supplementary material for: Contribution of Bone Marrow-Derived Hematopoietic Stem/Progenitor Cells to the Generation of Donor-Marker+ Cardiomyocytes In Vivo
Source: PLoS One. 2013 May 7;8(5):e62506. doi: 10.1371/journal.pone.0062506 (PMC3647070; doi:10.1371/journal.pone.0062506)
Supplement: Materials and Methods S1 — Detailed information on purification of donor BM cells, cell culture and differentiation assay of Lin−/lowCD45− cells, and histological analysis. (DOC) [file pone.0062506.s008.doc]

**Materials and Methods S1**

**Purification of donor BM cells.**

Single cell suspension of harvested BM cells was made in Dulbecco’s modified Eagle’s medium (DMEM) containing 1% fetal calf serum (FCS). Three ml of 1.5-2.5 x 10^8^ BM cells suspension loaded onto 3 ml of 1.0875 g/cm^3^ density separation media (Cedarlane) in 14 ml round-bottom tube (BD Falcon) was centrifuged at 1190 g for 25 minutes. MNCs were collected from the interface layer, diluted with PBS, and collected by centrifugation. Antibody labeling was preformed in PBS with 2% FCS for 30 minutes at room temperature. For separation of Lin^-/low^CD45^+^ cells and Lin^-/low^CD45^-^ cells and for enrichment of Lin^-/low^Sca-1^+^ cells, biotin-conjugated rat anti-mouse lineage antigen (CD3e, CD11b, B220, Gr-1, and TER-119) antibodies (BD Pharmingen) and sheep anti-rat IgG antibody-conjugated immunomagnetic beads (Dynal) were used to deplete mature hematopoietic cells from MNCs of GFP-transgenic or wild-type C57BL/6 mice. Phycoerythrin (PE)-conjugated anti-mouse CD45 or Sca-1 antibody (BD Pharmingen) and anti-PE immunomagnetic microbeads (Miltenyi Biotec) were used for magnetic cell sorting from Lin^-/low^MNCs. Lin^-/low^CD45^-^ cells separated from C57BL/6 mice were labeled with fluorescein isothiocyanate (FITC)-conjugated anti-CD29 antibody (BD Pharmingen), followed by flow cytometric analysis using a FACSCalibur (BD). For purification of GFP^+^Lin^-^Sca-1^+^c-Kit^+^ (LSK) cells and GFP^+^CD34^-^Lin^-^Sca-1^+^c-Kit^+^ (CD34^-^LSK) cells by fluorescence-activated cell sorting (FACS) as described [1], Lin^-/low^MNCs were enriched from GFP mice BM by using PE-Cy5-conjugated rat anti-mouse lineage antigen (CD3, CD4, CD8a, CD19, B220, and Gr-1) antibodies (Caltag) and sheep anti-rat IgG antibody-conjugated immunomagnetic beads. Lin^-/low^c-Kit^+^ cells were further enriched from Lin^-/low^MNCs by magnetic cell sorting using allophycocyanin (APC)-conjugated anti-mouse c-Kit antibody (BD Pharmingen), and anti-APC immunomagnetic microbeads (Miltenyi Biotec), followed by labeling with PE-cyanin-7 (PE-Cy7)-conjugated anti-mouse Sca-1 antibody (eBioscience) and biotin-conjugated anti-mouse CD34 antibody (RAM34; eBioscience), subsequently labeled with APC-Cy7-conjugated streptavidin (Caltag). For purification of GFP^+^Lin^-^Thy1.2^-^Sca-1^-/low^c-Kit^+/low^ hematopoietic progenitors, GFP^+^Lin^-^Thy1.2^-^IL-7Rα^-^Sca-1^-^c-Kit^+^ myeloid progenitors [including CMPs, GMPs, and megakaryocyte/erythrocyte progenitors (MEPs)] GFP^+^Lin^-^Thy1.2^-^IL-7Rα^+^Sca-1^low^c-Kit^low^ CLPs by FACS as described [2,3], Lin^-/low^Thy1.2^-/low^MNCs were enriched from GFP mice BM by using biotin-conjugated rat anti-mouse lineage antigen (CD3e, CD11b, B220, Gr-1, and TER-119) antibodies, biotin-conjugated rat anti-mouse Thy1.2 antigen antibody (eBioscience), and sheep anti-rat IgG antibody-conjugated immunomagnetic beads, followed by labeling with PE-conjugated anti-mouse IL-7Rα antibody (eBioscience), PerCP-cyanin-5.5 (PerCP-Cy5.5)-conjugated streptavidin (BD Pharmingen), APC-conjugated anti-mouse c-Kit antibody, PE-Cy7 conjugated anti-mouse Sca-1 antibody. For purification of GFP^+^Lin^-^IL-7Rα^-^Sca-1^-^c-Kit^+^FcγR^low^CD34^+^ CMP and GFP^+^Lin^-^IL-7Rα^-^Sca-1^-^c-Kit^+^FcγR^high^CD34^+^ GMP by FACS as described [2], Lin^-/low^IL-7Rα^-/low^MNCs were enriched from GFP mice BM by using PE-Cy5-conjugated rat anti-mouse lineage antigen (CD3, CD4, CD8a, CD19, B220, and Gr-1) antibodies, PE-Cy5-conjugated rat anti-mouse IL-7Rα antibody (eBioscience), and sheep anti-rat IgG antibody-conjugated immunomagnetic beads. Lin^-/low^IL-7Rα^-/low^c-Kit^+^ cells were further enriched from Lin^-/low^IL-7Rα^-/low^MNCs by magnetic cell sorting using APC-conjugated anti-mouse c-Kit antibody, and anti-APC immunomagnetic microbeads, followed by labeling with PE-conjugated anti-mouse FcγR antibody (BD), PE-Cy7-conjugated anti-mouse Sca-1 antibody, and biotin-conjugated anti-mouse CD34 antibody, subsequently labeled with APC-Cy7-conjugated streptavidin. All stained cells were resuspended in PBS containing 2% FCS and 1 μg/ml propidium iodide (PI), and passed through a cell strainer of 70-μm nylon (BD Falcon) before cell sorting. Single or 10 GFP^+^CD34^-^LSKs were sorted directly into individual wells of a U-bottom 96 well plate (BD Falcon) containing 100 μl PBS with 2% FCS by using a FACSAria automated cell deposition unit (BD). For higher numbers of cells, donor cells were sorted into 5 ml round-bottom tube (BD Falcon) containing 3 ml PBS with 2% FCS and collected by centrifugation. Purities of sorted cells were more than 95% by reanalysis in all cases.

**Cell culture and differentiation assay of Lin^-/low^CD45^-^ cells.**

Separated GFP^+^Lin^-/low^CD45^-^ cells and BM cells harvested from the recipients transplanted with GFP^+^Lin^-/low^CD45^-^ were cultured in DMEM containing 10% FCS, 50 units/ml penicillin (Gibco), and 50 μg/ml streptomycin (Gibco) in a 6 well culture plate (BD Falcon). Medium was half replaced every 3 to 4 days. Adherent cells were examined by using inverted research microscope (IX71, Olympus). In the culture of GFP^+^Lin^-/low^CD45^-^ cells, adherent cells were examined for their differentiation capacity using osteogenic or adipogenic induction medium [4]. Osteogenic induction medium containing dexamethasone, L-glutamine, ascorbate, and β-glycerophosphate (Cambrex) was used for osteogenic differentiation assay. Adipogenic induction medium containing insulin, L-glutamine, dexamethasone, indomethacin, 3-isobutyl-1-methyl-xanthin and adipogenic maintenance medium containing insulin, L-glutamine (Cambrex) were used for adipogenic differentiation assay. Alkaline phosphatase activity was determined by staining with mixed fast red violet solution with napthol solution (Chemicon) for identification of differentiated osteoblasts. Lipid vacuoles in the cytoplasm were visualized by Oil red O staining (Hokudo) for identification of differentiated adipocytes. In the culture of BM cells from GFP^+^Lin^-/low^CD45^-^ recipients, adherent cells were analyzed for the expression of hematopoietic antigens by immunostaining with anti-CD45 (DakoCytomation) or anti-CD11b antibody (BD Pharmingen), followed by subsequent staining with Cy3-conjugated secondary antibody (Jackson ImmunoResearch).

**Histological analysis.**

For hematoxylin and eosin (HE) stain and van Gieson stain, performed to observe histological changes following ventricular puncture, paraffin-embedded hearts were sectioned into 3-μm slices with a microtome (SM 2000R, Leica). For immunofluorescence staining, vibratome-sectioned cardiac slices (100-μm thickness) stained with primary and secondary antibodies were finally counterstained with DAPI (Vector Laboratories). Estimated number of recipient cardiomyocytes we examined was approximately 400,000 per a recipient mouse by repetitive count of cardiomyocytes in 40 contiguous heart sections of 4-6 week-old mice. Frequency of GFP^+^ cardiomyocytes in total of examined recipient cardiomyocytes was calculated as the number of GFP^+^ cardiomyocytes divided by 400,000 per a recipient mouse. In the transplantation of GFP^+^ BM cells into wild type C57BL/6 mice, cardiac sections were examined for the expression of 4 colors (DAPI, GFP, Cy3, and Cy5). In the transplantation of GFP^+^ BM cells into CFP-transgenic mice, cardiac sections were examined for the expression of 5 colors (DAPI, CFP, GFP, Cy3, and Cy5). The emitted fluorescence from cardiac sections of CFP-transgenic recipients stained with TnI-Cy3, Cx43-Cy5, and DAPI was detected at 10-11 nm interval between 417 and 749 nm wavelength in every 0.5 μm depths by using 458 nm Argon excitation laser (LSM510 META, Carl Zeiss). Linear unmixing analysis was employed to specifically detect GFP and CFP expressions as previously reported [5]. Briefly, the detected each fluorescence at 10-11 nm interval between 449 and 663 nm wavelength was calculated by the equation [S(λ)_SUM_ = Intensity x S(λ)_CFP_ + Intensity x S(λ)_GFP_] to determine the composition of GFP and CFP. The composition of GFP and CFP was examined simultaneously at multiple points of cardiomyocytes reconstructed in three-dimensional images. To analyze bone cortex, femurs of C57BL/6 recipient transplanted with GFP^+^Lin^-/low^CD45^-^ cells were fixed in paraformaldehyde, decalcified in ethylenediaminetetraacetic acid (EDTA), embedded in paraffin, and sectioned into 5-μm slices. Sectioned bone slices were stained with anti-GFP (Molecular Probes) and anti-CD45 (DakoCytomation) antibodies, subsequently stained with Cy3 or Cy5-conjugated secondary antibodies, and observed by confocal microscopy (LSM-GB200, Olympus).

**References**

1. Osawa M, Hanada K, Hamada H, Nakauchi H (1996) Long-term lymphohematopoietic reconstitution by a single CD34-low/negative hematopoietic stem cell. Science 273: 242-245.
2. Akashi K, Traver D, Miyamoto T, Weissman IL (2000) A clonogenic common myeloid progenitor that gives rise to all myeloid lineages. Nature 404: 193-197.
3. Kondo M, Weissman IL, Akashi K (1997) Identification of clonogenic common lymphoid progenitors in mouse bone marrow. Cell 91: 661-672.
4. Pittenger MF, Mackay AM, Beck SC, Jaiswal RK, Douglas R, et al. (1999) Multilineage potential of adult human mesenchymal stem cells. Science 284: 143-147.
5. Dickinson ME, Bearman G, Tille S, Lansford R, Fraser SE (2001) Multi-spectral imaging and linear unmixing add a whole new dimension to laser scanning fluorescence microscopy. BioTechniques 31: 1272, 1274-1276, 1278.
